# Emerging Role of Sirtuin 2 in Parkinson’s Disease

**DOI:** 10.3389/fnagi.2019.00372

**Published:** 2020-01-10

**Authors:** Yumei Liu, Yingying Zhang, Konghua Zhu, Song Chi, Chong Wang, Anmu Xie

**Affiliations:** ^1^Department of Neurology, The Affiliated Hospital of Qingdao University, Qingdao, China; ^2^Department of Neurology, The Eighth People Hospital of Qingdao City, Qingdao, China

**Keywords:** SIRT2, PD, Sirtuin, α-synuclein, tubulin, p53, inflammation, autophagy

## Abstract

Parkinson’s disease (PD), the main risk factor of which is age, is one of the most common neurodegenerative diseases, thus presenting a substantial burden on the health of affected individuals as well as an economic burden. Sirtuin 2 (SIRT2), a subtype in the family of sirtuins, belongs to class III histone deacetylases (HDACs). It is known that SIRT2 levels increase with aging, and a growing body of evidence has been accumulating, showing that the activity of SIRT2 mediates various processes involved in PD pathogenesis, including aggregation of α-synuclein (α-syn), microtubule function, oxidative stress, inflammation, and autophagy. There have been conflicting reports about the role of SIRT2 in PD, in that some studies indicate its potential to induce the death of dopaminergic (DA) neurons, and that inhibition of SIRT2 may, therefore, have protective effects in PD. Other studies suggest a protective role of SIRT2 in the context of neuronal damage. As current treatments for PD are directed at alleviating symptoms and are very limited, a comprehensive understanding of the enzymology of SIRT2 in PD may be essential for developing novel therapeutic agents for the treatment of this disease. This review article will provide an update on our knowledge of the structure, distribution, and biological characteristics of SIRT2, and highlight its role in the pathogenesis of PD.

## Introduction

Parkinson’s disease (PD) is one of the most common neurodegenerative diseases and is characterized by the loss of dopaminergic (DA) neurons in the striatum and the aggregation of Lewy bodies, the main component of which is α-synuclein (α-syn; de Oliveira et al., 2012; Satoh and Imai, 2014). The exact etiology of PD is still unclear, and aging is suggested to be one of the strongest factors for the progression of this disease (Collier et al., 2011; Dexter and Jenner, 2013; Pringsheim et al., 2014; Beilina and Cookson, 2016; Pellegrini et al., 2017). Abnormal aggregation, the change of microtubule dynamics, oxidative/nitrative damage, inflammation, and autophagy are recognized as vital physiological process which is associated with DA neuron degeneration in PD (Hirsch et al., 2012; Lynch-Day et al., 2012; Pellegrini et al., 2017). Many therapeutic approaches have aimed at increasing dopaminergic, while inhibiting cholinergic activity. The limited success of these approaches makes it essential to identify new therapeutic strategies. One such strategy may be targeted at aging-related deteriorations, as aging affects many pathophysiological processes involved in PD.

Sirtuins are conserved from bacteria to humans. There are seven human homologs, sirtuin1–7 (SIRT1–7). SIRT1 is ubiquitously expressed in all tissues including the brain, particularly in neurons (Ramadori et al., 2008). SIRT1 may play a protective role in PD. On the one hand, SIRT1 could suppress the aggregation of α-syn by activating molecular chaperones in animal and cell models of PD (Donmez et al., 2012). SIRT1 deacetylase activity mediates clearance of α-syn through light chain 3 (LC3) mediated autophagy to protect against PD pathology (Guo et al., 2016). Moreover, extracellular α-syn accumulation leads to mitochondrial dysfunction and a reduction of SIRT1 expression (Motyl et al., 2018). One the other hand, the upregulation, and activation of SIRT1 could activate peroxisome proliferator-activated receptor γ co-activator 1 (PGC-1α) to confer DA neuron protection against oxidative stress (Mäkelä et al., 2016). In addition, SIRT1 was shown to deacetylate histone residue H3K9 which is the p53 promoter, eventually leading to the reduction expression and protecting against apoptosis in SH-SY5Y cells (Feng et al., 2015). But no protection was observed in a 1-methyl-4-phenyl-1,2,3,6-tetrahydropyridine (MPTP)-induced PD model using SIRT1 transgenic mice (Kakefuda et al., 2009). Activating SIRT1 may seem to be beneficial for the organism against certain age-associated diseases. But the role of SIRT1 in PD needs to be further researched. Indeed, small compounds that could elevate the content of SIRT3 are protective against neuronal injury induced by 1-methyl-4-phenylpyridinium (MPP^+^) which is degraded from MPTP (Hu et al., 2014). Knock-out of SIRT3 significantly exacerbated nigrostriatal neuron death by MPTP (Liu et al., 2015; Zhang et al., 2016).

Sirtuin 2 (SIRT2), part of the family of sirtuins, belongs to class III histone deacetylases (HDACs) and is most abundantly found in the cortex, striatum, hippocampus, and spinal cord (Maxwell et al., 2011). The enzymatic activity of SIRT2 is dependent on nicotinamide adenine dinucleotide (NAD^+^), and it not only catalyzes the deacetylation of histone substrates, but also that of non-histone substrates (Landry et al., 2000). SIRT2 thus regulates a large spectrum of physiological processes such as genome stability, mitosis, nutrient metabolism, aging, mitochondrial function, autophagy, myelination, apoptosis, antioxidant mechanisms and cell motility (North and Verdin, 2007; Maxwell et al., 2011; Liu et al., 2014, 2017; Braidy et al., 2015; Gomes et al., 2015; Fourcade et al., 2017). Recent studies have indicated that SIRT2 is implicated in several aging-related neurodegenerative diseases, and the fact that its expression increases not only with age but also in PD models suggests its key role in this particular disease (Harting and Knöll, 2010; Maxwell et al., 2011; Poulose and Raju, 2015; Sun et al., 2018). Here, we present a brief review article of the structure, distribution and biological characteristics of SIRT2. We then summarize the current literature and provide a comprehensive analysis of the role of SIRT2 in PD, and its potential as a therapeutic target for the treatment of this disorder.

## SIRT2: Structure, Distribution, and Biological Characteristics

SIRT2 is one isoform of class III sirtuins, which differ from class I, II and IV in that their catalytic activity requires NAD^+^ as a cofactor for catalysis. SIRT2 catalyzes the deacetylation of both histone and non-histone substrates (Cen et al., 2011). This enzyme has a highly conserved catalytic core domain consisting of about 275 amino acid residues. The catalytic core comprises two staple parts: a large domain and a small domain. Unlike other sirtuins, the large Rossmann-fold domain, which is a typical NAD^+^ binding site, is formed by a central β-sheet surrounded by seven α-helices. The small domain contains three anti-parallel β-sheets (β4–6), one α-helix (α9) and a Zn^2+^ cation, which is coordinated by four cysteine residues (Parenti et al., 2015). Although zinc ions do not directly participate in catalytic activities, they are necessary to ensure normal activity of the enzyme (Min et al., 2001). The small domain has the most variable regions, suggesting that they may play important roles in regulating key properties, such as substrate specificity, and may also be binding-sites for sirtuin-selective modulators. The two domains are joined by a number of flexible loops, termed cofactor-binding loop, and together form a large groove. The conjunctive groove contains the NAD^+^-binding site, which is conserved among all sirtuins. The catalytic core substrate-binding pocket can be divided into three subdomains: the adenine ribose moiety of NAD^+^ is bound in site A, the nicotinamide ribose moiety is bound in site B, while site C, located deep inside the pocket and containing the catalytic center, binds nicotinamide during catalysis (Finnin et al., 2001). The C-and N-terminal extensions of SIRT2 differ from other sirtuins and play a crucial role in its subcellular localization and distribution (Chang et al., 2002).

SIRT2 not only possesses stronger deacetylation activity, but also features adenosine diphosphate (ADP) nucleic acid transferase activity, demyristoylase activity and mediates long adipose chain diacylation (Landry et al., 2000; Teng et al., 2015). The deacetylation reaction is the transfer of the substrate’s acetyl group to the ADP-ribosyl moiety of NAD^+^, whereby one NAD^+^ molecule splits into nicotinamide (NAM) and O-acetyl ADP ribose (OAADPr; Shimizu et al., 2016); Similar to other family members of sirtuins, SIRT2 is widely distributed throughout the body, and is found to be particularly broadly expressed in metabolically active tissues and organs such as liver, prostate, pancreas, kidney, and adipose tissue in mice. Recent research has found that SIRT2 levels are distributed more in the central nervous system (CNS), especially in the cortex, striatum, hippocampus and spinal cord (Maxwell et al., 2011). A study demonstrated that expression levels of the SIRT2 in the Substantia Nigra pars compacta (SNpc) remain relatively unaltered with PD development, indicating the potential of its targeting in PD patients (Harrison et al., 2018). Within the CNS, SIRT2 is expressed in the neurites and growth cones of neurons (Pandithage et al., 2008), as well as in oligodendrocytes (OLs), the myelinating cells of the CNS. Li et al. (2007) found that overexpression of SIRT2 inhibits OL differentiation, together indicating that this enzyme plays a crucial role in CNS diseases.

SIRT2 regulates microtubule function, cell cycle, oxidative stress, autophagy, and neuroinflammation, all of which are recognized as prominent processes in the pathogenesis of PD (Zhao et al., 2010b; Maxwell et al., 2011; de Oliveira et al., 2012; Liu et al., 2014; Kida and Goligorsky, 2016). The level of SIRT2 increases in models of PD, suggesting it may have a significant effect also in the human disease (Wang et al., 2015; Guan et al., 2016; Sun et al., 2018). SIRT2 is mainly localized to the cytoplasm and deacetylates cytoplasmic proteins, such as the main component of microtubules (MT): α-tubulin. Its main site of action is lysine 40, thereby affecting intracellular transport and cell integrity (North et al., 2003). Within the G2/M phase of mitosis, SIRT2 is transferred from the cytoplasm to the nucleus where it deacetylates histone H4 at lysine 16, thus reducing the level of H4K16 acetylation, which in turn decreases chromatin condensation and facilitates DNA replication, but the specifical role in the pathogenesis of PD is not clear (Vaquero et al., 2006). In addition, SIRT2 also shuttles to the nucleus in response to cellular stress and is capable of deacetylating the forkhead box class O (FOXO) family of transcription factors, which are pivotal in a myriad of physiological processes (Wang et al., 2007; Pais et al., 2013; Akbulut et al., 2015). Under oxidative stress, SIRT2 releases FOXO1, which is then being acetylated and able to bind to ATG7, eventually contributing to autophagy in cancer (Zhao et al., 2010a). SIRT2 is also associated with nuclear transcription factor kappa B (NF-κB), which plays an important role in gene regulation related to aging and inflammation (Rothgiesser et al., 2010). Inhibition of SIRT2 causes a decrease in cytoplasmic p53 expression, subsequently promoting autophagy through the resulting increase in the acetylation level of this tumor suppressor (Sun et al., 2018). In addition, SIRT2 acts in maintaining mitochondrial biology (Liu et al., 2017).

The role of SIRT2 in the pathogenesis of neurodegenerative diseases is controversial. SIRT2 overexpression promotes neurodegeneration, which may be attributed to enhanced deacetylation of tubulin to impair microtubule stability in neurons (Outeiro et al., 2007). In a mouse model of Huntington’s disease, the inhibition of SIRT2 led to a decreased accumulation of polyglutamine in the N-terminus of neurons (Qiu et al., 2010). Furthermore, the expression of SIRT2 was found to exacerbate α-syn toxicity in models of PD, whereas the inhibition of SIRT2 led to an increase in the survival of neuronal cells (de Oliveira et al., 2017). It was also found that SIRT2 expression can decrease neuronal cell death in animal models treated by MPP^+^. Thus, SIRT2 may have different functions depending on the context. In the following sections, the role of SIRT2 in the most important PD-associated processes will be discussed.

## The Role of SIRT2 in Parkinson’s Disease

### SIRT2 Increases α-syn Aggregation and Toxicity

Lewy bodies, eosinophilic inclusion bodies that appear in the nigrostriatal system, are a typical pathological feature of PD (Spillantini et al., 1997). The most important component of the Lewy body is α-syn. Within the normal CNS, α-syn is abundant, mainly in the pre-synaptic membranes and cytoplasm of the striatum, neocortex, olfactory bulb, hippocampus, SN, thalamus and amygdala. α-syn is a protein of 140 amino acids consisting of three parts: the N-terminus (amino acids 1–60), which is highly conserved in the synuclein family; a central portion which is highly hydrophobic and is thought to underlie the aggregation-prone nature of the protein; and an acidic C-terminal tail (amino acids 96–140) which is highly charged and is a site subject to post-translational modifications (Krumova et al., 2011). Previous studies have shown that the acetylation of α-syn occurs at the N-terminus and interacts with the liquid membrane. This N-terminal acetylation improves α-helical folding induced by the membrane, reducing the aggregation of α-syn (Bartels et al., 2014; Theillet et al., 2016). α-syn is highly soluble under normal physiological conditions and plays a crucial role in regulating the size of the vesicle pool, vesicle transport, docking of vesicles with the pre-synaptic membrane, and DA biosynthesis. Furthermore, α-syn plays a protective role in oxidative stress, thus reducing DA toxicity (Quilty et al., 2006). Soluble α-syn is degraded by the ubiquitin-proteasome system (UPS) and chaperone-mediated autophagy (CMA), whereas insoluble α-syn tends to form aggregates which inhibit the system of degradation and induce toxicity-a key factor in the pathogenesis of PD (Zhang et al., 2008). The abnormal aggregation of α-syn leads to the formation of insoluble inclusion bodies, simultaneously reducing the level of soluble, functional α-syn. This, in turn, causes impairment of UPS function and acceleration of mitochondrial dysfunction, increases in sensitivity to oxidative stress, and enhances DA transporter-mediated toxicity, thereby promoting cell death and contributing to the development of PD pathogenesis (Sharma et al., 2006; Buttner et al., 2008).

**Figure 1:**
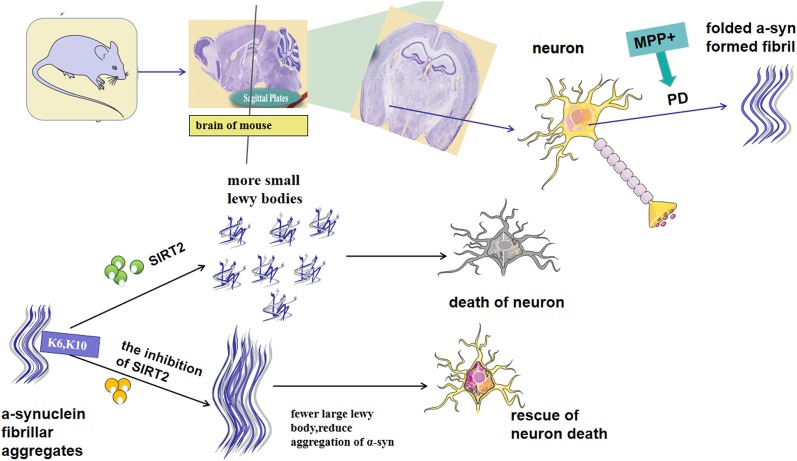
Possible mechanism for Sirtuin 2 (SIRT2) to regulate α-synuclein (α-syn), the aggregation of which is the key characteristic of Parkinson’s disease (PD) pathogenesis. SIRT2 affects the conformation of α-syn through deacetylation at K6 and K10 in the conserved N-terminal region. Besides, the expression of SIRT2 can lead to an increase in small Lewy bodies, whereas its inhibition increases α-syn levels. SIRT2 inhibition has been demonstrated to protect neural cells from α-syn-mediated neurotoxicity. In contrast, other studies have reported an increase in α-syn following inhibition of SIRT2.

SIRT2 has been reported to exacerbate α-syn toxicity in models of PD (de Oliveira et al., 2017). In contrast, a different study found that inhibition of SIRT2 increases the aggregation of α-syn and aggravates toxicity. These findings demonstrate that the role of SIRT2 in this context needs further investigation. It is known that SIRT2 affects the conformation of α-syn through deacetylation at K6 and K10 in the conserved N-terminal region, thus rendering it more prone to aggregation (de Oliveira et al., 2017; Figure 1). Additionally, SIRT2 may also regulate the main clearance pathway of α-syn by interfering with the alkaline phosphatase (ALP; Sampaio-Marques et al., 2012). The same study also showed that increased α-syn acetylation and knockdown of SIRT2 led to a reduction in the aggregation of α-syn, and therefore decreased toxicity (de Oliveira et al., 2017). Outeiro et al. (2007) found that the expression of SIRT2 induces the formation of small Lewy bodies, increasing neurotoxicity induced by α-syn. Moreover, arabidopsis guanylate kinase 2 (AGK2)-and adenylate kinase 1 (AK1)-mediated inhibition of SIRT2 increases the volume of α-syn inclusion bodies in cells transfected with labeled α-syn, and the enlargement of such inclusion bodies are reported to reduce their toxicity (Outeiro et al., 2007). AK7-mediated inhibition of SIRT2 was found to ameliorate α-syn toxicity and provide neuroprotection in models of PD (Chen et al., 2015). SIRT2 levels in the brain increase with aging, which is accompanied by a decrease in acetylated α-syn (Maxwell et al., 2011; de Oliveira et al., 2017). This, in turn, leads to the production of smaller Lewy bodies, interferes with the clearance of α-syn and decreases levels of its soluble version, thus increasing overall α-syn-mediated toxicity and loss of DA neurons (Figure 1). α-syn aggregation can also be induced by oxidative stress, which is aggravated by AGK2-mediated inhibition of SIRT2 (Singh et al., 2017). A study found that increased mitophagy activity, mediated by the regulation of ATG32 by SIRT2, is an important phenomenon linked to SNCA-induced toxicity during aging (Rubinsztein et al., 2011; Sampaio-Marques et al., 2012). While the majority of the literature has reported that the expression of SIRT2 can aggravate α-syn-induced neurotoxicity, the precise functional relationship between SIRT2 and α-syn remains elusive. Further research will be needed to better understand the role of SIRT2 in this context and help to clarify its potential as a target for therapeutic intervention in PD.

### SIRT2 Exacerbates Oxidative Damage

Oxidative stress is considered a key factor contributing to the degeneration of DA neurons in PD. There is still controversy about the specific role of SIRT2 in oxidative stress in the context of this disease. While some studies have reported that SIRT2 aggravates oxidative damage, others have found the opposite. In animal models of PD, MPTP, 6-hydroxy-dopamine (6-OHDA), diquat and rotenone were found to produce reactive oxygen species (ROS; Perier et al., 2007). The elevation of malondialdehyde (MDA), lipids and cholesterol hydroperoxide in the substantia nigra (SN) of PD patients reflect the increase of oxidative stress in PD, caused by an imbalance in the production and clearance of ROS (Dexter et al., 1989). Rats treated by rotenone exhibit a range of motor symptoms, which may be exacerbated by elevated SIRT2 in response to rotenone (Wang et al., 2015). Selective SIRT2 inhibition *via* adenylate kinase 7 (AK7) significantly diminishes striatal DA depletion and improves behavior abnormalities in rotenone-treated aging rats (Wang et al., 2015). Similar results were obtained in experiments in which mice were treated with MPTP, substantiating the role of SIRT2 in aggravating oxidative damage (Guan et al., 2016). DA neurons are sensitive to oxidative stress based on the high content of iron and polyunsaturated fatty acids, leading to a greater generation of ROS and an increased rate of DA neuron death due to mitochondrial dysfunction and neuroinflammation (Sanders and Timothy Greenamyre, 2013; Mackeh et al., 2014; Navarro-Yepes et al., 2014; Rivas-Arancibia et al., 2015; Guo et al., 2018).

**Figure 2:**
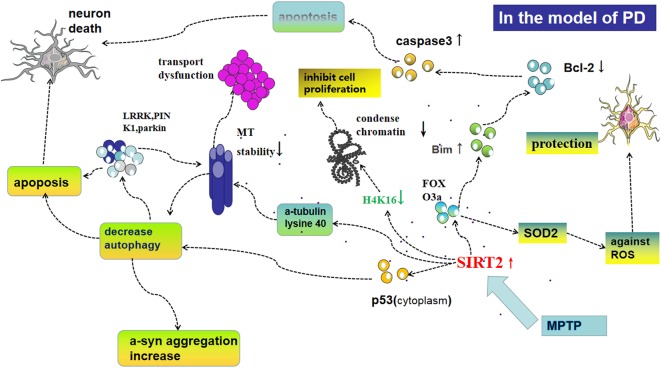
Possible mechanisms of SIRT2 for regulating oxidative stress, autophagy, and the function of microtubules (MT), all of which play an important role in the pathogenesis of PD. Following MPP^+^-treatment in cells or methyl-4-phenyl-1,2,3,6-tetrahydropyridine (MPTP)-injection in mice, the expression of SIRT2 increases levels of cytoplasmic p53 and subsequently decreases autophagy, which could lead to apoptosis and aggregation of PD-associated proteins such as leucine-rich repeat kinase 2 (LRRK2), PTEN-induced kinase 1 (PINK1), and parkin, which decrease the stability of MT and further cause apoptosis. Furthermore, SIRT2 is able to deacetylate α-tubulin at lysine 40, thereby declining the stability of MT and leading to a shortening of neurites. Deacetylation of FOXO3a by SIRT2 leads to activation of pro-apoptotic protein-Bim, then inhibiting the anti-apoptotic activity of Bcl-2 and activating caspase-3, initiating, initiating apoptosis in mitochondria, thus resulting in fewer cells producing dopamine (DA) in the SN only after MPP^+^-treatment in cells or MPTP-injection in mice. SIRT2 acts at H4K16, which in turn decreases chromatin condensation and facilitates DNA replication, but the specifical role in the pathogenesis of PD is not clear. FOXO3a also regulates SOD2 to protect neural cells against reactive oxygen species (ROS).

Furthermore, oxidative stress is closely related to apoptosis, another physiological process SIRT2 is implicated in. In this context, SIRT2 deacetylates FOXO3a, thus activating pro-apoptotic protein-Bim, inhibiting the anti-apoptotic activity of Bcl-2, activating caspase-3, initiating apoptotic neuronal death (Liu et al., 2014; Li et al., 2016; Figure 2). This eventually results in fewer cells producing DA in the SN only after MPP^+^-treatment in cells or MPTP-injection in mice; and deletion or silencing of SIRT2 prevents neuronal cells death (Liu et al., 2014; Figure 1). The inhibition of SIRT2 also has protective effects *in vitro* as well as in a *Drosophila* model of PD (Outeiro et al., 2007). Nie et al. (2014) found that AGK2-mediated SIRT2 inhibition protects differentiated PC12 cells from toxic damage caused by H_2_O_2_ and that silencing SIRT2 decreased ROS production after H_2_O_2_ treatment. Another study found that microRNA-7 (miR-7) inhibits SIRT2, causing a decrease in RelA expression and a relieve of NF-κB suppression, consequently protecting against MPP^+^-induced cell death (Choi et al., 2014). Contrasting results from other groups demonstrate that SIRT2 can also be beneficial to the survival of DA neurons. In SH-SY5Y cells for example, SIRT2 shuttles to the nucleus and rescues cells from oxidative damage by deacetylation of FOXO3a, thereby increasing expression of FOXO3a targets such as SOD2 and counteracting the effects of ROS. In addition, when SH-SY5Y cells are treated by diquat or rotenone, AGK2-mediated inhibition of SIRT2 was also shown to promote cell death (Singh et al., 2017).

### SIRT2 Decreases Microtubule Stability

Cytoskeletal homeostasis is crucial for the development and function of the nervous system. MT, one of three main components of the cytoskeleton, are polar cylindrical polymers of α/β-tubulin heterodimers (Janke, 2014; Coles and Bradke, 2015). They are also an integral part of the spindle, centrosomes and specialized cellular structures like flagella and cilia (Subramanian and Kapoor, 2012). In neurons, MTs also play important roles during the morphological transitions that occur during neuronal development, such as neurite initiation, migration, polarization, and differentiation (Kapitein and Hoogenraad, 2015). MT defects cause a wide range of nervous system abnormalities and several human neurodevelopmental disorders have been linked to altered microtubule-mediated processes (Kapitein and Hoogenraad, 2015). In addition, SIRT2 is overexpressed during mitosis, affecting the cell cycle and its activity has been found to be deregulated in PD (Dryden et al., 2003; Garske et al., 2007; Inoue et al., 2009). Mounting evidence indicates cytoskeletal dysfunction to be one factor contributing to PD pathogenesis. A number of proteins implicated in PD such as α-syn, parkin, PTEN-induced kinase 1 (PINK1), and leucine-rich repeat kinase 2 (LRRK2) have been shown to bind tubulin and regulate MT stability, highlighting the involvement of MT in the pathogenesis of this disease (Alim et al., 2004; Yang et al., 2005; Weihofen et al., 2009; Dagda et al., 2014; Godena et al., 2014; Law et al., 2014). α-syn is MT-associated protein (MAP; Esteves et al., 2019). It was demonstrated that the MT network and MT-dependent trafficking are impaired upon overexpression of α-syn (Lee et al., 2006). The structure and function of MT are also regulated by other post-translational modifications such as acetylation of α-tubulin at lysine 40, which enhances MT stability and increases the transport efficiency of cargo proteins along the MT (Dompierre et al., 2007; Creppe et al., 2009; Solinger et al., 2010). This modification is also associated with other biological processes such as fibrillar hair depolymerization, cell migration, and autophagy, and is known to influence cellular stress, inflammation and viral responses (Ishiguro et al., 2011; Misawa et al., 2013; Sabo et al., 2013; Mackeh et al., 2014; Wang et al., 2014). In sporadic PD brains, a decrease in the level of acetylated MT could be caused by a change in mitochondrial metabolism, which is related to the activation of SIRT2 (Esteves et al., 2018).

In neurons, recent studies have shown that MT acetylation is essential for normal neuronal development and function (Creppe et al., 2009; Solinger et al., 2010). The degree of acetylation is balanced by the controlled activity of acetylases and deacetylases. One of the main acetylases is acetyltransferase 1 (TAT1), also known as MEC-17 (Akella et al., 2010; Kalebic et al., 2013). The activity of deacetylation is mediated by two enzymes, the NAD-independent histone deacetylase 6 (HDAC6) and the NAD^+^-dependent deacetylase SIRT2 (Hubbert et al., 2002; Matsuyama et al., 2002; North et al., 2003). The activity of SIRT2 is mainly affected by the intracellular NAD^+^ content, which correlates with tubulin acetylation (Skoge et al., 2014). Conversely, NAD depletion leads to the activation of HDAC6, thereby increasing the deacetylation of tubulin (Skoge et al., 2014). These findings indicate that SIRT2 and HDAC6 act on different subunits of α-tubulin in their deacetylation and acetylation activities, respectively (Hubbert et al., 2002; Matsuyama et al., 2002; North et al., 2003; Skoge and Ziegler, 2016). Importantly, it was shown that in sporadic PD patient-derived cells, when NAD^+^ metabolism is altered, SIRT2 is activated, causing the level of acetylation of α-tubulin to diminish. Consequently, by inhibiting SIRT2 activity, the levels of acetylated α-tubulin are increased, thereby improving MT dynamics *via* enhanced α-syn/tubulin binding. The activation of SIRT2 or HDAC6 could increase the tubulin deacetylation and induce MT loss stability and depolymerization (Esteves et al., 2019).

SIRT2 mediates MT deacetylation, thus impairing the integrity and causing a shortening of neurites, a key feature of PD pathogenesis. 6-OHDA is a neurotoxin that can cause PD-like symptoms in models of this disease. It is transported into neuronal cells by DA re-uptake transporters and generates ROS through various mechanisms, eliciting an oxidative damage response and inducing a decrease in the rate of MT growth (Patel and Chu, 2014). In addition, 6-OHDA alters the subcellular localization of certain transcription factors, leading to an alteration in gene transcription and decreasing the survival of midbrain neurons (Chalovich et al., 2006). At least to an extent, the above-mentioned effects of 6-OHDA may be mediated by its inhibition of SIRT2 activity and the consequent reduction in the rate of deacetylation, independently of NAD^+^ levels (Patel and Chu, 2014). In animal models of PD, MPP^+^ specifically acts on the kinetic system of MT synthesis, thereby impairing this process and promoting apoptosis of DA neurons, confirming the importance of a functioning MT in the context of PD (Cappelletti et al., 2005). In sporadic PD patient-derived cells, it was observed that selective inhibition of SIRT2 restored the levels of tubulin acetylation, reducing the ratio of free/polymerized tubulin and improving MT-mediated transportation (Esteves et al., 2018). In addition, AK7-mediated inhibition of SIRT2 results in increased levels of acetylated α-tubulin, attenuating the loss of striatal DA and nigral TH^+^ neurons and improving motor function. In summary, the above-described findings suggest an important role of SIRT2 in regulating MT function in the context of PD (Chen et al., 2015; Wang et al., 2015; Guan et al., 2016).

### SIRT2 Aggravates Neuroinflammation

A growing number of studies have shown that PD progression is characterized by chronic inflammation-induced DA neuron degeneration within the SN (Schapira, 2013). Increased microglial and astrocyte activation, cyclooxygenase-2 (COX-2), pro-inflammatory cytokines and nitric oxide (NO) levels have been reported in many toxic animal models of PD (Noelker et al., 2013). In this context, MPTP causes chronic inflammation and progressive neurotoxicity, but the underlying mechanisms remain all not clear (Fox and Brotchie, 2010). It has been suggested that inflammation-associated oxidative stress and cytokine-dependent toxicity are at least in part responsible for the loss of DA neurons in PD (Frankola et al., 2011). Activated microglia produce large amounts of superoxide and NO, causing oxidative/nitrative stress and neurotoxicity in the CNS. These cells also release pro-inflammatory cytokines such as tumor necrosis factor-alpha (TNF-α), or neurotoxic glutamate, leading to the degeneration of DA neurons in MPTP models of PD which deteriorates with aging (García-Domínguez et al., 2018; Yao and Zhao, 2018). The death of DA neurons also results in the release of harmful molecules such as oxidized proteins, lipids and DNA, which in turn activate microglia and sustain a pro-inflammatory environment (Block et al., 2007). Together, these findings suggest that microglial activation is a key event in neuroinflammation in PD (Frank-Cannon et al., 2009).

It was shown that SIRT2 is required for microglial activation induced by lipopolysaccharide (LPS) and that SIRT2 inhibition decreases microglial activation and alleviates neuroinflammation, ultimately decreasing DA neuron death. A study also showed that siRNA-mediated knockdown of SIRT2 had a similar effect on microglia and led to a decrease in the production of NO and inflammatory cytokines such as TNF-α and interleukin 6 (IL-6), indicating that SIRT2 is critical for microglial activation induced by LPS (Chen et al., 2015). SIRT2 is required for LPS-induced activation of BV2 microglia. A recent study provided evidence that inhibition of SIRT2 by AGK2 impairs microglia survival and decreases the ATP levels of microglia-mediated by poly (ADP-ribose) polymerase (PARP) activation, which is a known mediator of programmed necrosis (Li et al., 2013). In addition, SIRT2 deacetylates p65 at Lys310, regulating the expression of NF-κB-related genes (Deeb et al., 2010). Therefore, SIRT2 could promote inflammation and neuronal cell death by activating transcription of NF-κB (Amigo and Kowaltowski, 2014). The effect of SIRT2 on the survival of microglia under resting and activated conditions is unclear. AGK2 has been shown to reduce the survival rate of basal small cells, whether it would have a favorable or adverse effect on PD. In conclusion, SIRT2 is able to induce microglial activation, thereby promoting inflammation in the CNS, which may have important implications in the pathogenesis of PD.

### SIRT2 Impairs Autophagy

Autophagy is a highly conserved mechanism of lysosome-mediated protein and organelle degradation that plays a crucial role in maintaining cellular homeostasis. The process of autophagy is categorized into micro- and macro-autophagy and CMA, the latter plays an important role in the occurrence and development of PD. There is an ever-enlarging body of evidence that suggests that several processes implicated in PD pathogenesis converge on impaired CMA function, including α-syn accumulation, mitochondrial dysfunction, and oxidative stress. Furthermore, CMA is related to genes that are the cause of PD such as LRRK2, UCH-L1 and PAPR7 (Kabuta et al., 2008; Orenstein et al., 2013; Wang et al., 2016). In addition, an impairment of autophagic activity leads to the deposition of a variety of harmful PD-related proteins besides α-syn such as LRRK2, PINK1, Parkin, and ATP13A2 which are related to PD and trigger apoptosis (Venderova and Park, 2012; Martinez-Vicente, 2015; Figure 2). What’ more, PINK1 detects mitochondrial dysfunction and then signals Parkin to ubiquitinate specifically the damaged mitochondria to instigate their removal by autophagy, indicating PINK1 and parkin regulate autophagy activity together (Pickrell and Youle, 2015). Previous studies have identified transcription factors p53 and FOXO, as well as histones H3 and H4 as sirtuin-regulated targets, suggesting that SIRT2 may interfere with the expression of protective genes and thereby contribute to a loss of neurons. Indeed, it has been reported that the tumor suppressor p53 is a major deacetylation substrate of SIRT2 (van Leeuwen et al., 2013). An increase in p53 acetylation *via* SIRT2 inhibition reduces cytoplasmic p53 levels, thus blocking the inhibitory effect of cytoplasmic p53 on autophagy (Sun et al., 2018; Figure 2). As in PD models the expression of p53 is elevated, inhibition of SIRT2 rescues autophagy function, demonstrating its crucial role in this context.

The activity of autophagy is also closely associated with MT function and aggregation of α-syn. As mentioned above, the clearance of α-syn is regulated by autophagy, and a dysfunction of this process can lead to an aggregation of this protein, which is recognized as the underlying mechanism in the development of sporadic PD (Tofaris et al., 2011). Furthermore, the fusion of autophagosomes with lysosomes requires acetylated MT, and MT activity mediates the formation of autophagosomes and the sorting and transport of cargo (Xie et al., 2010). AK1-mediated inhibition of SIRT2 was shown to restore MT stabilization and improve autophagy. In addition, α-syn-mediated neurotoxicity in several PD models is partly due to deacetylation of α-tubulin by SIRT2 (Outeiro et al., 2007). Together, SIRT2 may present a key target in restoring autophagy function, which could have a promising potential in therapeutic intervention in PD (Sampaio-Marques et al., 2012).

## Conclusion

In summary, SIRT2 not only acts on histones, but also on a variety of non-histone proteins to regulate various physiological activities such as inflammation, cell cycle, stress, et cetera. Some researches implicated that the expression of SIRT2 could damage the survival of neuronal cells. Under stress, it acts on FOXO3a and increases its deacetylated degree, activating Bim and caspase-3, initiates apoptosis in the mitochondrial pathway, resulting in fewer cells producing DA in the SN. In cells transfected with α-syn, SIRT2 inhibitors may increase the volume of α-syn inclusion bodies, reduce the number and their toxic effects on nerve cells. Under stress, SIRT2 acts on a-tubulin, resulting in a decrease in the degree of a-tubulin acetylation. The stability is weakened, causing changes in kinetics and shortening of axons, resulting in the death of nerve cells; SIRT2 promotes the development of PD by acting on MT, α-syn, inflammation, and autophagy, which need more researches to support. In addition, cell culture studies demonstrate that AGK2 could inhibit the activity of SIRT 2 and result in neuroprotection in degenerating dopaminergic neurons. The inhibition of SIRT2 such as miR-212-5p promotes autophagy by decreasing the deacetylation of cytoplasmic p53 expression. SIRT2 inhibitors can reduce the death of DA neurons. SIRT2 inhibitors have neuroprotective effects, there may exist other SIRT2 inhibitory molecules that, we have not discovered. They delay or prevent the progression of PD by acting on certain pathological processes of PD pathogenesis. By studying these inhibitory small molecules, It may provide a new strategy for the treatment of PD, and more researches and experiments are needed to explore in the future. On the contrary, some studies found the SIRT2 could rescue cells from oxidative damage and AGK2-the inhibition of SIRT2 could aggravate the cellular death under oxidative stress. For the role of SIRT2 in PD is controversial for now at least although the most of studies implicate the expression of SIRT2 could lead to damage of neuronal cells in PD and the inhibition of SIRT2 could decrease the death of neuron cells. Further studies would be essential to estimate the role in PD. Meanwhile, the relationship between the function of MT, the aggregation of α-syn, autophagy is not clear, requiring more researches. Increasing studies have researched the inhibition of SIRT2, which is demonstrated to decrease the death of cells in PD, exploring the potential therapy for treating PD. 5-[(3-amidobenzyl)oxy]nicotinamides which presents a new class of SIRT2 inhibitors is a potential therapy for PD (Ai et al., 2016). However, the interactions between synuclein aggregation, inflammation, oxidative stress, MT, autophagy, and apoptosis are unclear and require further study. 3-[(2-methoxynaphthalen-1-yl)methyl]-7-[(pyridin-3-ylmethyl)amino]-5,6,7,8-tetrahydrobenzo[4,5]thieno[2,3-d]pyrimidin-4(3H)-one (ICL-SIRT078), a substrate-competitive SIRT2 inhibitor is recovered as a candidate neuroprotective agent in an *in vitro* PD model.

## Author Contributions

AX planned the study. YL, SC, KZ, CW, and YZ analyzed the data and edited the manuscript. YL wrote the manuscript.

## Conflict of Interest

The authors declare that the research was conducted in the absence of any commercial or financial relationships that could be construed as a potential conflict of interest.
